# ScalableDigitalHealth (SDH): An IoT-Based Scalable Framework for Remote Patient Monitoring

**DOI:** 10.3390/s24041346

**Published:** 2024-02-19

**Authors:** Hisham Alasmary

**Affiliations:** Department of Computer Science, College of Computer Science, King Khalid University, Abha 61421, Saudi Arabia; alasmary@kku.edu.sa

**Keywords:** autoscaling, AWS cloud, digital health, edge computing, Internet of Things (IoT), Kubernetes, remote health monitoring

## Abstract

Addressing the increasing demand for remote patient monitoring, especially among the elderly and mobility-impaired, this study proposes the “ScalableDigitalHealth” (SDH) framework. The framework integrates smart digital health solutions with latency-aware edge computing autoscaling, providing a novel approach to remote patient monitoring. By leveraging IoT technology and application autoscaling, the “SDH” enables the real-time tracking of critical health parameters, such as ECG, body temperature, blood pressure, and oxygen saturation. These vital metrics are efficiently transmitted in real time to AWS cloud storage through a layered networking architecture. The contributions are two-fold: (1) establishing real-time remote patient monitoring and (2) developing a scalable architecture that features latency-aware horizontal pod autoscaling for containerized healthcare applications. The architecture incorporates a scalable IoT-based architecture and an innovative microservice autoscaling strategy in edge computing, driven by dynamic latency thresholds and enhanced by the integration of custom metrics. This work ensures heightened accessibility, cost-efficiency, and rapid responsiveness to patient needs, marking a significant leap forward in the field. By dynamically adjusting pod numbers based on latency, the system optimizes system responsiveness, particularly in edge computing’s proximity-based processing. This innovative fusion of technologies not only revolutionizes remote healthcare delivery but also enhances Kubernetes performance, preventing unresponsiveness during high usage.

## 1. Introduction

The Internet of Things (IoT) serves as a global network interconnecting users, devices, and services collectively termed “Things”. It is designed to facilitate information exchange and enable monitoring, control, and calibration of devices on a worldwide scale [1]. With its technological foundations, IoT finds diverse applications including smart cities, traffic management, industry optimization, and crucially, healthcare [2].

In the healthcare paradigm, the IoT plays a pivotal role, particularly in the context of personalized wellness plans, chronic disease management, and elderly care [3]. It effectively empowers the monitoring of patients’ health statuses, ensuring adherence to prescribed treatments and medications under the guidance of medical professionals [4]. Key components of the IoT-enabled healthcare framework encompass medical devices, vital sign sensors, and diagnostic instruments, collectively forming a dynamic ecosystem [5].

Within this framework, IoT enables advanced functionalities such as Computer Assisted Diagnostics (CAD) and data-driven decision support systems, amplifying the capabilities of healthcare providers [6]. Moreover, IoT-driven healthcare addresses the unique needs of the elderly, individuals with disabilities, and those residing in remote areas with limited access to medical facilities [7,8].

IoT-enabled healthcare’s transformative potential extends to substantial benefits in cost reduction, enhanced quality of life, and comprehensive patient assessment [9]. This technology fosters improved interactions between healthcare professionals, patients, attendants, clinics, and medical institutions, ultimately nurturing a holistic healthcare environment [10]. Furthermore, through IoT-enabled healthcare, proactive measures are taken to detect chronic and infectious diseases at early stages, curbing their societal impact such as COVID-19 [11]. Real-time emergency responses are also significantly bolstered, ushering in a new era of timely decision-making [12].

The emergence of edge computing has not only revolutionized computational resources but has also ushered in a transformative shift in various industries, providing efficient and low-latency services [13,14]. However, this technological shift brings new challenges. Network latency becomes a crucial concern as computational processes move closer to the edge, requiring careful management for optimal performance. Ensuring reliability poses another challenge, given the distributed nature of edge systems with varying device capabilities. Security concerns are heightened due to the decentralized architecture, demanding robust measures to protect data integrity. Addressing these challenges is essential to establish a resilient edge computing framework [15,16].

As industries increasingly adopt agile microservices, replacing traditional architectures, they also leverage containerized resources at the edge to optimize system efficiency [17]. This approach resonates particularly well within the realm of IoT-driven healthcare, where the seamless integration of microservices and containerization can amplify the monitoring and management of patient health statuses through applications [18].

However, as these advancements unfold, the importance of latency-sensitive workloads cannot be overlooked, especially when considering autoscaling strategies. While the industry explores reactive, proactive, and machine learning-based approaches for scaling decisions, the intrinsic relationship between latency and autoscaling efficacy is sometimes underestimated [19]. In the context of IoT-enabled healthcare, this oversight could compromise the real-time monitoring and emergency response capabilities that form the core of its transformative potential. Hence, understanding and addressing the latency factor becomes crucial to ensure the optimal performance of edge systems deployed in healthcare scenarios.

By integrating the cutting-edge concepts of edge computing and IoT, healthcare practitioners, and technology experts can collectively build a robust ecosystem that not only monitors patients’ well-being but also ensures swift and efficient decision-making. In the subsequent sections, a more extensive examination is conducted to explore the synergistic interplay between IoT-driven healthcare and edge computing, highlighting their combined potential to revolutionize the healthcare landscape.

Several remote patient monitoring systems have been proposed in the literature; however, these solutions do have their limitations. One of the challenges is the lack of scalability, in the architectures used by many of these systems. With the increasing demand for patient monitoring, these architectures struggle to handle the growing volume of patient data in real time, which leads to bottlenecks and system failures. Also, it is crucial to address the issue of responsiveness in real-time healthcare data transmission and processing. Delays in receiving and analyzing data can impact the timeliness of interventions, thereby affecting the ability of the system to offer insights promptly. As a result, these scalability and responsiveness drawbacks negatively impact patient monitoring effectiveness, potentially affecting patient outcomes and the quality of the care provided. A novel ScalableDigitalHealth (SDH) architecture is introduced in this work to effectively tackle the aforementioned challenges in remote patient monitoring by integrating IoT/wearable devices and micro application autoscaling into a robust healthcare framework. The core objective is to optimize SDH capabilities for remote patient monitoring. The architecture seamlessly manages data acquisition, computation, and analysis, meeting complex IoT performance criteria. It adapts to varying demands at the edge and end users by dynamically scaling monitoring applications. The major contributions and novelty of this work are as follows:

- A scalable IoT-based architecture for remote health monitoring, incorporating a layered framework for efficient data management, and a dynamic latency-driven microservice autoscaling strategy.
- An innovative microservice autoscaling algorithm in edge computing, which adjusts the number of pods based on endpoint latency, determined by dynamic latency thresholds and enhanced by the integration of custom metrics and a Prometheus adapter.
- A more sophisticated and efficient system that optimizes Kubernetes clusters by adjusting pod counts according to latency metrics, dynamically adapting to varying workloads, enhancing system responsiveness, and preventing unresponsiveness during high usage.

This paper is organized as follows: In Section 2, a comprehensive review of related work is presented. Section 3 outlines the proposed SDH framework for remote patient monitoring, including the design, implementation, and integration of hardware/software systems. In Section 4, the experimental setup and validation of real-time health monitoring data on the AWS platform are detailed in Section 5, highlighting the attained results and performance gains. Finally, Section 6 concludes this paper by summarizing the contributions and outlining potential directions for future research in this domain.

## 2. Related Work

IoT refers to a networked system comprised of integrated sensors, actuators, controllers, and communication devices that facilitate the collection, transmission, and processing of data remotely. It has a wide range of applications in the healthcare system, mainly for remote healthcare, treatment, and rehabilitation in some cases [20]. It does so by attributing all active resources into a network to monitor health status such as detection, observation, and teleoperation using the Internet [21].

### 2.1. Remote Patient Monitoring

There have been quite a few contributions reported in the literature; for instance, in [22], the authors proposed a system for healthcare monitoring that utilizes bio-medical sensors and gateway communication. Despite showcasing advanced techniques, the system’s performance is commendable; however, the absence of robust security mechanisms is a significant limitation. Nerella et al. [23] presented an IoT-enabled healthcare architecture for monitoring critical patients in the ICU. Their system provides real-time recommendations and alerts based on vital sign fluctuations. Although the architecture has enhanced ecological parameters and acquired necessary software and hardware resources, the authors acknowledge the need for additional sensors to measure pressure and weight, which somewhat limits its comprehensive applicability.

Wearable devices have steered new possibilities for biomedical IoT applications by facilitating data acquisition and transmission for further analysis. Categorizing portable patient observation systems into groups based on sensor types offers a structured approach. The wearable sensors are capable of recording various parameters such as heartbeat, pressure, photoplethysmography (PPG), and radio frequency (RF). While these wearables exhibit promising potential for monitoring and intervention, the practicality and reliability of such devices in different scenarios warrant further scrutiny [24]. In this regard, Majumder et al.’s approach [25] employs a multi-sensor wearable device for real-time cardiac arrest analysis, utilizing machine learning algorithms and signal processing techniques. This demonstrates a step forward in accurate diagnosis and timely alerts; however, the specific algorithms and methodologies employed should be critically evaluated to ensure their effectiveness across diverse cases. The insertion of sensors into clothing for continuous monitoring, as proposed by Brezulian et al. [26], presents an interesting perspective. The utilization of nasal thermistor sensors for respiratory rate determination adds a novel dimension to monitoring techniques. The reliance on temperature changes for respiration calculation may introduce challenges in accuracy, particularly in non-ideal conditions. Wang et al.’s innovative solution [27] for measuring breathing rates in asthma patients introduces watermarking and signal enhancement for secure data transmission. While the concept is intriguing, the practical implementation and potential drawbacks of watermarking in a medical context deserve careful consideration. Despite the promising potential of IoT-enabled devices, several studies [28] reveal concerns regarding reliability, traceability, and security within the IoT framework for commercial blood pressure measurement. The use of small, easily integrated sensors is highlighted as essential, but the feasibility of maintaining accuracy and reliability while downsizing sensors remains a critical concern.

### 2.2. Application Scaling at the Edge

The landscape of autoscaling microservices in both traditional cloud and modern edge computing contexts has gained significant attention due to the inherent constraints posed by limited resources such as CPU, memory, and storage. The complexity is compounded by the heterogeneity of edge devices, necessitating dynamic resource adjustments to ensure system stability and performance [29]. The deployment of containers, as well as the adoption of different scalability paradigms (vertical, horizontal, and hybrid scaling) across various computational layers in edge environments, have aimed to address these challenges. However, it is important to examine the existing approaches critically.

While the Horizontal Pod Autoscaler (HPA) has found its place in cloud environments, its applicability and effectiveness in the context of microservice scaling for edge computing remain questionable [30]. The intricate nature of edge scenarios demands a broader consideration of metrics due to the diverse resource landscape across heterogeneous devices.

Past research endeavors have leveraged performance metric tools like Prometheus to facilitate application development, deployment, and autoscaling in edge computing. Novel contributions have sought to address container scaling and workload balancing specific to the edge. Casalicchio’s study on performance measures for autoscaling, though claimed to improve Quality of Service (QoS), must be scrutinized for its actual impact, especially in real-world, dynamic scenarios [31]. Similarly, Ahmad et al.’s categorization of container scheduling approaches, while comprehensive, does not necessarily guarantee their effectiveness in addressing real-world bottlenecks and pitfalls [32].

The adaptation of HPA and its variants for autoscaling microservices in edge computing raises concerns about their efficacy and responsiveness. Nguyen et al.’s comparison of Autoscaler responses to Kubernetes and Prometheus metrics warrants a closer look to assess the practical significance of their findings [33]. The DHPA algorithm by Jiang et al. acknowledges the limitations of standard HPA, yet its potential drawbacks, especially in more complex edge scenarios, remain under-explored [34]. The dynamic multi-level autoscaling approach proposed by TaheriZadeh et al. seems promising, but its comparative performance with other state-of-the-art methods requires rigorous testing and validation [35].

Machine learning-based approaches for autoscaling have gained traction; however, a critical evaluation of their practicality is needed. The Proactive Pod Autoscaler introduced by Ju et al. claims to forecast workloads and scale proactively, but the feasibility of real-time forecasting in edge contexts must be examined [19]. Buchaca et al. in [36] coined the concept of a perceptron-based prediction mechanism which appears promising, but the generalizability of their approach across different edge use cases should be assessed. Similarly, the RL-based approach by Rossi et al. may excel in cloud settings, but its applicability and performance at the edge demand thorough investigation [37].

In summary, while the literature presents a multitude of autoscaling approaches for microservices in edge computing, several critical aspects warrant further investigation. The existing methodologies often lack thorough testing in diverse, real-world edge scenarios, and the applicability of machine learning-driven techniques must be evaluated under the constraints of latency, resource scarcity, and dynamic workloads. A more rigorous and critical assessment is essential to ensure that proposed autoscaling mechanisms translate into tangible benefits in the intricate and real-time edge computing-based healthcare framework.

## 3. Proposed Methodology

**Figure 1:**
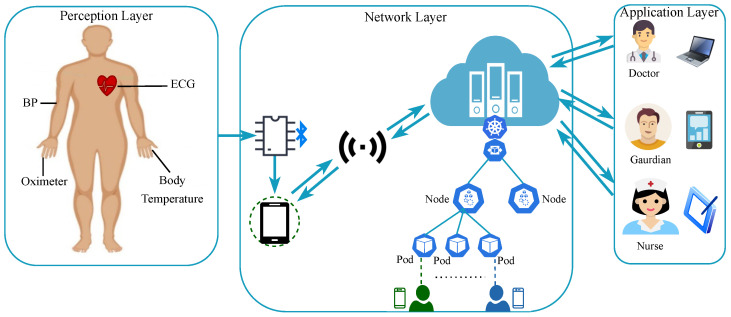
System model of the proposed scalable digital health framework.

We provide a comprehensive overview of our proposed methodology encompassing architecture, key components, and necessary procedures, detailing how it achieves the development of latency-aware autoscaling-based remote patient monitoring. Our architecture, depicted in Figure 1, seamlessly combines three essential functional layers: the perception layer, the network layer, and the application layer. The perception layer connects patients, sensors, and IoT devices, gathering vital health data using IoT modules. These data are then transmitted through the network to the AWS cloud. The network layer manages data transmission, secure storage, and dynamic processing within the cloud ecosystem. Responsiveness is enhanced through latency-based Kubernetes Horizontal Pod Autoscaling, improving the user experience of our mobile app. The application layer provides a user interface for medical professionals, clinicians, patients, and caregivers. This empowers them to access patient data, respond in real time, and interact with the system. Further details about each layer and its components are provided in the subsequent sections.

### 3.1. Perception Layer

The IoT devices and sensors at the perception layer record vital health data from the patient’s body, transmitting them to the cloud, as depicted in Figure 1. This layer initially gathers data from sensors, aggregating them in a Microcontroller Unit (MCU), and then sends them to the smartphone via Bluetooth. The smartphone further transfers data to the cloud via a gateway.

Equipped with an Arduino microcontroller, the perception layer integrates body temperature, ECG, blood pressure, and oxygen saturation sensors linked to a smartphone app. However, managing abundant sensor-generated data at the perception layer is challenging because, for example, incorporating more sensors is hindered by data volume, low-power smartphones struggle with extensive data, and data processing expenses rise due to network congestion. To address this, our solution employs edge computing. As depicted in Table 1, redundant data are evident in pulse and temperature sensor readings taken every four milliseconds. Variable sample rates is assigned to sensors based on importance, allowing more frequent readings for significant sensors like ECG. Moreover, microcontroller-level edge computation identifies and discards redundant sensor data, minimizing unnecessary data transmission.

Efficient IoT sensor data transfer for processing begins by transmitting them to the smartphone app developed using the Flutter framework. Flutter, an open-source UI toolkit that supports both iOS and Android, facilitates this communication via Bluetooth. To manage data load, our approach involves secondary edge processing. This entails the smartphone analyzing data and determining the necessity of immediate cloud transmission; otherwise, it is stored locally. For instance, transmitting safe body temperature readings (97–99 °F or 36.11–37.22 °C) immediately is unnecessary. Local processing also eases the load on the cloud gateway. The smartphone facilitates module connections, controlling data flow. Through distinct sensor frequencies and redundant data filtering on the microcontroller and smartphone, our system adeptly manages high data volumes, creating space for additional sensors.

### 3.2. Network Layer

This layer, also known as the cloud layer, facilitates data management, collection, storage, and processing. The data received from the perception layer are forwarded to the cloud storage after initial processing. This layer consists of several modules, including gateways, communication interfaces, AWS cloud, Kubernetes engine, and more.

In the AWS cloud, the system can receive data from the smartphone app using either MQTT, a lightweight publish–subscribe protocol for IoT devices, or the lambda event service. The proposed system selects the event service due to its efficient data processing, microservices integration, and robust authentication.

Once data are received on the event server, the system authenticates and verifies them using JSON Web Token (JWT), ensuring data source credibility. If needed, an extra layer of security through encryption is applied. After verification, the system evaluates readings to identify critical situations needing immediate medical attention. In such cases, health representatives and caregivers are notified via the mobile application, or else the data are sent to storage.

The framework classifies cloud storage as Relational Data Storage (RDS) and non-relational storage. RDS instances hold patient records and recent analyses, while non-relational storage, managed through a simple storage service (S3), stores vast IoT module data, retrievable upon demand.

The cloud processes and analyzes the streamlined data from the mobile app, utilizing simulated intelligence, ML algorithms, and visualization tools, enabling advanced diagnoses and medical decision support for physicians. The system showcases primary visual effects and comments to demonstrate framework effectiveness.

To optimize Kubernetes clusters, the system dynamically adjusts pod counts based on latency metrics. The proposed algorithm calculates the necessary pod count adjustments (*P*) for a target latency value ($L_{target}$), considering active pods, max and min number of pods, and latency threshold values. A waiting period (τ) is introduced for stability. The algorithm excels in adapting to varying workloads, enhancing Kubernetes performance.

In terms of the decision-making algorithm (Algorithm 1), the Threshold Latency Value is collected and measured using the Custom Metrics API and Prometheus. The system considers a situation of many Kubernetes nodes (swarm) and nodes with different capabilities, where pods can generate different latencies.
**Algorithm 1** Latency-based Horizontal Pod Autoscaling with Custom Metrics API**Require:**$L_{\mathrm{target}}$: Target latency value
1:ActivePods: Set of currently active pods2:MinPods: Minimum number of pods3:ScalingThreshold: Threshold for scaling (e.g., percentage increase in latency)4:CustomMetrics: Custom Metrics API for collecting and measuring latency values5: 6:Initialize Custom Metrics API: CustomMetrics.initialize()7:MaxPods = None8:**while True:**9:       L={}10:     **for** pod **in** ActivePods:11:       $L_{i}=\mathrm{getThresholdLatencyValue}\left(pod\right)$12:       $L.\mathrm{append}(L_{i})$13:      **end for**14:      total Latency=∑(L)15:      $averageLatency=\frac{total\;Latency}{\mathrm{ActivePods}.\mathrm{length}}$16:      **if** MaxPods is None:17:         MaxPods = determineMaxPods()18:      $latencyChange=\frac{\left(averageLatency-L_{target}\right)}{L_{target}}\times 100$19:      **call** AggregateLatenciesAcrossNodes()20:      **call** CalculateSeparateLatencyThresholdsForEachNodeOrPod()21:      **call** TakeIntoAccountDifferencesInCapabilitiesAcrossNodes()22:      **if** latencyChange>ScalingThreshold **and** len(ActivePods) < MaxPods:23:         $P=\lceil \mathrm{ActivePods}.\mathrm{length}\times \frac{latencyChange}{ScalingThreshold}\rceil$24:         P=min(P,MaxPods−ActivePods.length)25:      **elif** latencyChange<−ScalingThreshold **and** len(ActivePods) > MinPods:26:         $P=\lceil \mathrm{ActivePods}.\mathrm{length}\times \frac{-latencyChange}{ScalingThreshold}\rceil$27:         P=min(P,ActivePods.length−MinPods)28:      **else:**29:         P=030:      **end if**31:      **if** P>0:32:         **call** ScaleUp(*P*)33:      **elif** P<0:34:         **call** ScaleDown(−P)35:      **end if**36:      **call** wait(τ)

The network layer facilitates Application-to-Network layer communication via API gateways, linking lambda functions to gateways using JSON Web Tokens (JWTs) for Application layer authorization. Upon successful authentication, specific requests trigger relevant service invocation.

The system utilizes the Custom Metrics API to empower monitoring tools like Prometheus to leverage application-specific metrics for the HPA controller. Network traffic metrics are applied to improve latency and response time. The Custom Metric API metrics encompass application statistics housed in Prometheus’ time series database and measurements from a collector integrated into Kubernetes Custom Metrics API.

The objective is to scale pods based on endpoint latency, achieved through the adoption of the Prometheus adapter via a Kubeapps Hub helm chart. The system contributes an HPA that adjusts deployment scaling when the average response time surpasses a set threshold, such as 50 ms.

The algorithm dynamically adjusts pod numbers based on latency, optimizing system responsiveness as multiple users interact simultaneously. It efficiently scales pods to align with observed latency, a methodology well-suited for edge computing’s proximity-based processing. The framework maintains the application’s responsiveness, providing timely healthcare services and adapting effectively to varying workloads and network conditions, enhancing remote patient monitoring.

### 3.3. Application Layer

**Figure 2:**
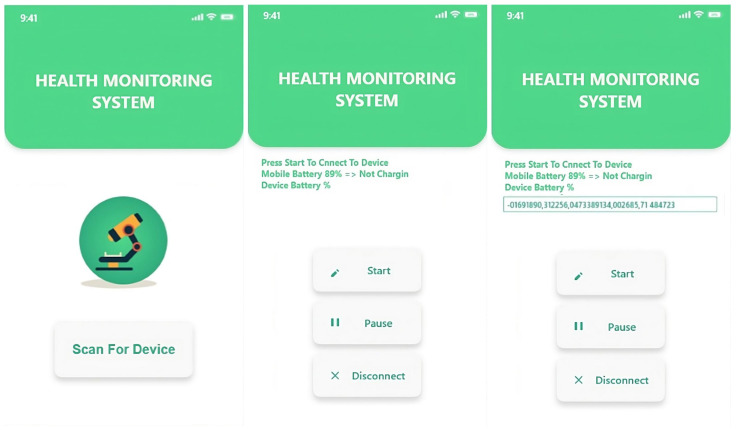
IoT devices registration via Flutter-based mobile app.

The application layer serves as the bridge for doctors, health workers, and guardians to use the system together. It includes web-based dashboards, physician, and guardian smartphone apps, as illustrated in Figure 2. Through these interfaces, users access real-time patient information. Medical staff may use it to visualize data and prompt alerts. This layer acts as the link between the system and doctors, enabling real-time patient health updates and healthcare data insights. The guardian app provides real-time patient health updates and alerts for immediate attention. The dashboard empowers healthcare professionals to monitor and diagnose patients, while also facilitating cloud-based analysis and quick guardian notifications.

## 4. Experimental Setup

The proposed scalable framework for remote patient monitoring using a custom-built solution that integrates IoT components and AWS cloud services is implemented. This systematic approach ensures real-time data transmission, storage, and access for the IoT-based remote health monitoring system. To provide a better understanding of the technical aspects of the prototype, the principal technical specifications of the electronic elements and modules used are summarized in Table 2.

**Figure 3:**
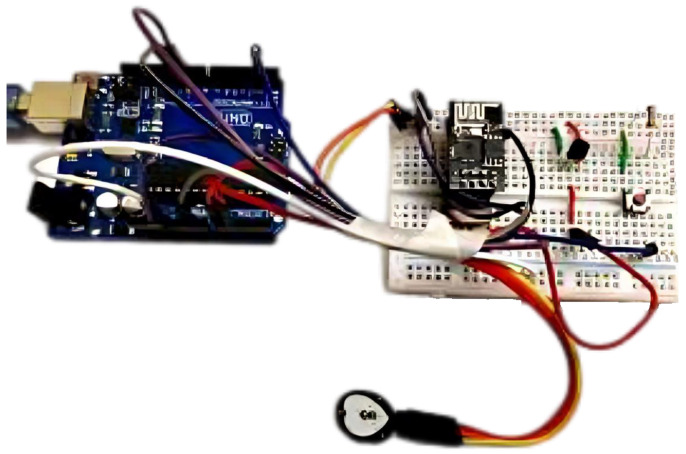
Hardware setup.

The central microcontroller unit, Arduino UNO Board, orchestrates various sensors and actuators, including the AD8232 ECG Sensor, LM35 Temperature Pulse Sensor, blood pressure sensor, and MAX30100 Pulse Oximeter. These components are meticulously configured (as illustrated in Figure 3) for precise data acquisition, enabling unique identifiers (Card IDs) for differentiating patients and using the HC05 Bluetooth module operates at a frequency of 2.4 GHz for secure communication with the Android app.

The ESP32 module is integrated with the Arduino UNO Board for IoT data transmission, collecting sensor data and securely transmitting them to the AWS cloud platform. The proposed system selects the event service due to its efficient data processing, microservices integration, and robust authentication. The AWS cloud services are leveraged for data storage, management, and authentication, enabling real-time data transmission, storage, and access for the IoT-based remote health monitoring system.

In addition, latency-based metrics into a remote patient monitoring Android application using a Prometheus client are incorporated, focusing on enabling Kubernetes to function effectively in a large-scale edge computing scenario with latency-aware Pod autoscaling. This experimental setup investigates the implications of these metrics in a microservice environment while connecting Kubernetes and Prometheus capabilities.

## 5. Results and Discussion

This section provides a detailed discussion on the outcomes of the proposed SDH framework after rigorous experimentation. The experiments were conducted in a controlled laboratory environment using a Kubernetes cluster (version 1.18.0) comprising a single Master node and three worker nodes. In this setup, the data accumulated through the IoT module including vital signs such as heart rate, blood pressure, and temperature were subsequently transmitted to the cloud. Intelligent insights and recommendations including real-time push notifications were generated at the cloud as a result of data analysis. Each monitoring session lasted 6 h, with a total of 10 sessions conducted to assess the performance and validity of the proposed novel framework over time. The evaluation focused on simulations in the laboratory settings analyzing the functionality and scalability. To evaluate the effectiveness of the Latency Aware HPA, the impact on Pod generation was analyzed using the latency custom metric. The Master node was equipped with a 4-core CPU and 8 GB RAM configuration, while each worker node had a 4-core CPU and 4 GB RAM configurations.

To replicate edge nodes within the proposed scalable remote patient monitoring framework and to keep track of latency metric values, multiple edge nodes were activated at different time stamps with the remote patient monitoring application deployed on worker nodes, accessible to edge nodes via the NodePort service.

**Figure 4:**
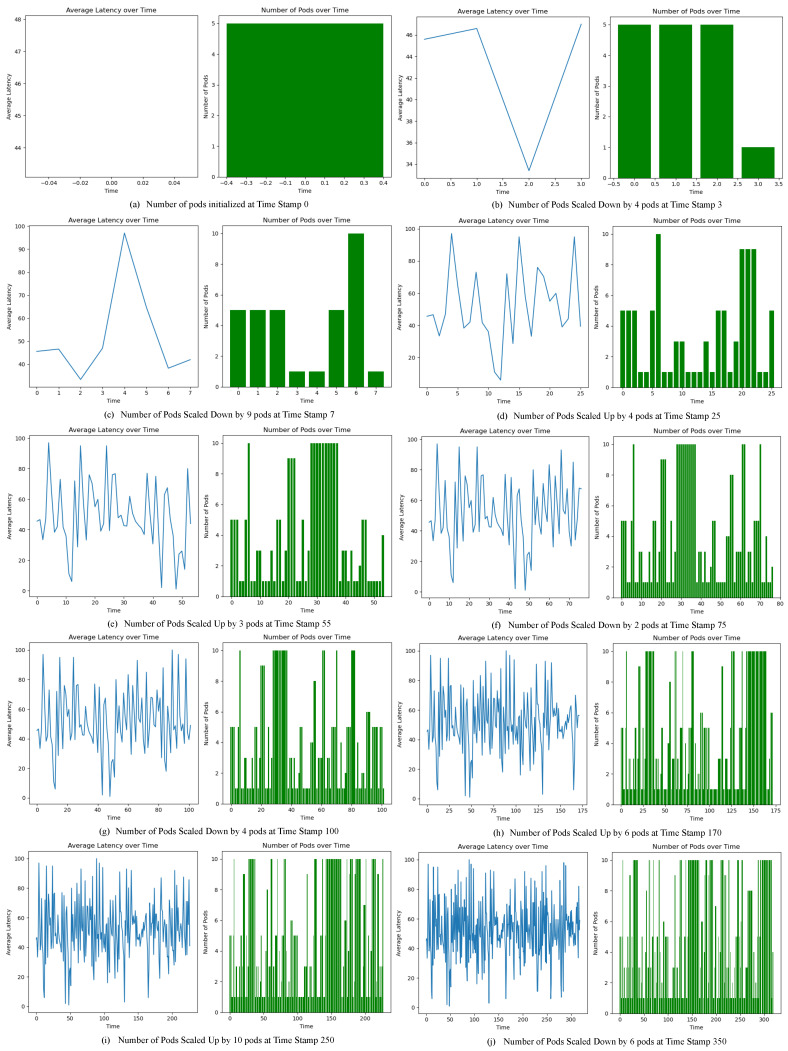
Average latency fluctuations and scaling decisions at multiple time stamps.

In the context of latency-aware scalability and pod adjustments, significant insights emerge from the observations, as depicted in Figure 4. The average latency values are captured and plotted as a continuous line graph, whereas the number of pods with respect to time stamps is shown in the green color bar plot. In Figure 4a, at time stamp 0 a fixed number of five pods were initialized. After a few minutes, edge nodes were activated to launch the smartphone application and subsequently a rise in the average latency values was observed, as depicted in Figure 4b. As a result, four pods were automatically added and consequently a drop in the average latency value could be seen. In order to further validate the effectiveness of the proposed scalable framework, two of the active edge nodes were deactivated and as a result a drop in the average latency values was observed and hence the framework scaled down nine pods at time stamp 7, as depicted in Figure 4c. The fluctuations in the average latency values were closely monitored, and at time stamp 25 another screenshot of the system was captured representing the addition of four extra pods, as seen in Figure 4d.

The effect of constant number of active edge nodes was also examined, as can be seen in Figure 4e; in the time stamp window around 30 and 36, the number of pods remained constant at the value 10, highlighting a very minor change in the average latency values. The experiment carried out over 6 h was closely monitored in 10 sessions and system performance in terms of scaling up (pod addition), scaling down (pod deletion), and no change (pod remain constant) was evaluated at different time stamps, as depicted in Figure 4a through Figure 4j.

Collectively, the critical analysis of these scenarios underscores the algorithm’s dynamic adaptability in the face of varying latency dynamics. The observations illustrate its capacity to strike a delicate equilibrium between accommodating performance and sustaining the stability of the underlying system.

Moreover, to validate the effectiveness of the proposed SDH framework, an extensive comparative analysis was carried out, bench-marking SDH against other state-of-the-art remote healthcare solutions. Through this comprehensive assessment, the proposed SDH framework for remote patient monitoring stands out as a robust and versatile solution, demonstrating excellent performance based on key features and services, as detailed in Table 3.

## 6. Conclusions and Future Work

In response to the rising demand for remote patient monitoring, particularly among vulnerable populations like the elderly and mobility-impaired, this study introduced the groundbreaking “ScalableDigitalHealth” (SDH) framework. By integrating smart digital health solutions with latency-aware edge computing autoscaling, this research has provided an innovative approach to remote healthcare. Leveraging IoT technology and application autoscaling, SDH enables the real-time tracking of critical health parameters, anticipated to change the dynamics of healthcare delivery by ensuring accessibility, cost-efficiency, and rapid responsiveness to patient needs. In the perspective of future research directions, some of the open issues need further exploration. One direction is enhancing the predictive analytic capabilities within the SDH framework. This may be achieved by integrating advanced machine learning algorithms that will empower the system to detect early signs of health deterioration and proactively alert healthcare providers. Additionally, a user-centric research is also vital, involving usability studies and user experience assessments to tailor the SDH framework to diverse patient demographics. By continually advancing such integrated healthcare solutions, patient-centric care will go beyond geographical barriers and will empower both patients and healthcare providers.

## Figures and Tables

**Table 1:** Temperature and pulse data collected using the IoT module.

Temp (F)	Temp (C)	Pulse	Time
80	26.67	87	10 April 2023 16:20:10
80	26.67	87	10 April 2023 16:20:12
80	26.67	87	10 April 2023 16:20:16
80	26.67	87	10 April 2023 16:20:20
80	26.67	87	10 April 2023 16:20:23
80	26.67	87	10 April 2023 16:20:27
80	29.44	85	10 April 2023 16:20:31
82	27.78	85	10 April 2023 16:20:34
84	28.89	85	10 April 2023 16:20:38
86	30.00	85	10 April 2023 16:20:42
87	30.56	85	10 April 2023 16:20:45
88	31.11	85	10 April 2023 16:20:49
89	31.67	85	10 April 2023 16:20:53
90	32.22	85	10 April 2023 16:20:56

**Table 2:** Technical specifications of electronic elements and modules.

Component	Technical Specifications
Arduino UNO Board	16 MHz clock speed, 32 KB of SRAM, 1 KB of EEPROM, and USB connectivity
AD8232 ECG Sensor	3-lead ECG sensor, 50 Hz noise rejection, and 2.5–3.3 V power supply
LM35 Temperature Pulse Sensor	0–150 °C temperature range, 0.25 °C sensitivity, and 3–5 V power supply
Blood Pressure Sensor	0–255 mmHg pressure range, I2C interface, and 5 V power supply
MAX30100 Pulse Oximeter	Pulse rate and SpO2 measurements, I2C interface, and 1.8–5.5 V power supply
HC05 Bluetooth Module	2.4 GHz frequency range, 2 Mbps bitrate, and RS232 interface
ESP32 Module	Dual-core 32-bit CPU, Wi-Fi and Bluetooth connectivity, and 3.3 V power supply
AWS Cloud Services	Amazon Web Services (AWS) IoT Core, AWS Lambda, AWS DynamoDB, and AWS CloudWatch
Flutter-based Mobile App	Open-source UI toolkit, supports iOS and Android, and Wi-Fi connectivity
Prometheus Client	Open-source monitoring and alerting toolkit, supports client libraries and integrations, and various frequency ranges for metric collection

**Table 3:** A comparative analysis of health monitoring systems featuring various capabilities.

References	Features						
	Monitoring Capabilities	Real Time	Remote Configuration	Scalability	Communication and Alerts	Personalized Recommendations	Medicine Reminder
Ref. [38]	Heart Chronic Disease	✓	✕	✓	✕	✕	✕
Ref. [39]	Total Knee Arthroplasty	✓	✕	✕	✓	✓	✕
Ref. [40]	Activities of Daily Living (ADLs)	✓	✕	✕	✓	✕	✕
Ref. [41]	Body Temperature, Heartbeat, ECG	✓	✕	✕	✓	✕	✕
Ref. [42]	ADLs, ECG, Fall Detection	✓	✕	✕	✓	✕	✕
Ref. [43]	Heart Disease, Monitoring	✓	✓	✓	✓	✕	✕
Ref. [44]	Pulse, Body Temperature, Heart Rate, Oxygen Saturation	✓	✕	✕	✓	✕	✕
Proposed SDH	Body Temperature, ECG, Blood Pressure, Oxygen Saturation	✓	✓	✓	✓	✓	✓

## Data Availability

The proposed dataset is private data and will be available upon request for research purposes.
